# INSL3 Expression in Leydig Cells is a Biomarker for Immunocastration in Boars: Transcriptional Evidence

**DOI:** 10.1111/andr.70136

**Published:** 2025-10-21

**Authors:** Nina Batorek Lukač, Martin Škrlep, Klavdija Poklukar, Petra Ferjan, Volker Stefanski, Gregor Fazarinc, Milka Vrecl

**Affiliations:** ^1^ Agricultural Institute of Slovenia Ljubljana Slovenia; ^2^ Veterinary Faculty, Institute of Preclinical Sciences University of Ljubljana Ljubljana Slovenia; ^3^ University of Hohenheim Stuttgart Germany

**Keywords:** alternatives to castration, INSL3, pig, response to immunocastration, testicular mRNA expression

## Abstract

**Background:**

Study aimed to obtain insights into physiological responses to immunocastration in pubertal boars by evaluating effects of alternative vaccination protocols and identifying a reliable immunocastration biomarker.

**Objectives:**

It was hypothesized that the timing of gonadotropin‐releasing hormone (GnRH) suppression by immunocastration differentially affects reproductive function, as reflected by testicular histology and expression of selected genes related to testicular function, steroid metabolism, and Leydig cell differentiation and function.

**Materials and Methods:**

Effects of three vaccination protocols on antibody titers, testicular histomorphology, and mRNA expression in immunocastrates slaughtered 4, 8, or 12 weeks (IC‐4, IC‐8, and IC‐12, respectively; *n* = 6 per group) after booster were compared with entire males (EMs; *n* = 6). Principal component analysis and hierarchical clustering were used to evaluate individual responses and to identify an immunocastration biomarker(s). The selected biomarker was also validated on samples from previous studies.

**Results:**

All immunocastrated boars reacted immunologically to vaccination (increased GnRH antibody titer; *p* < 0.004). There was an increased nucleus‐to‐cytoplasm ratio in Leydig cells (*p* < 0.033) and decreased testosterone concentration (*p* < 0.041) in IC‐4 compared with EM, whereas values in IC‐8 and IC‐12 were intermediate. Hierarchical clustering differentiated immunocastrates with low testosterone (IC‐LT; median 0.56 ng/mL) and high testosterone (IC‐HT; median 7.04 ng/mL). In IC‐LT, expression levels of FSHR and ESR2 were higher than in IC‐HT, and those of LHCGH, STAR and INSL3 were lower compared with IC‐HT and EM, whereas expression of ESR1 and HSD17β7 was lower in IC‐LT and IC‐HT compared with EM (fold change > 1.5 and *p* < 0.05).

**Conclusion:**

There were variable responses to immunocastration in pubertal boars subjected to three vaccination protocols. Suppression of testicular function was most pronounced in boars slaughtered 4 weeks after the booster, whereas progressive recovery occurred in some boars 8 and 12 weeks after the booster. Irrespective of vaccination protocol, INSL3 mRNA expression was a reliable immunocastration biomarker.

## 1 Introduction

Immunocastration suppresses androgens and has been available in the European Union since 2009; it was developed as an alternative to surgical castration of piglets [1]. Hypogonadism is achieved by active immunization against hypothalamic gonadotropin‐releasing hormone 1 (GnRH) with an initial vaccination and subsequent booster that trigger production of anti‐GnRH antibodies that block the hypothalamic–pituitary–gonadal axis, suppressing testicular function within 2–4 weeks (wk) after the booster [2, 3, 4]. Most available data on immunocastration refer to the standard vaccination protocol which involves administering first dose before puberty (usually between the age of 8–12 week), and the booster 4–6 week before slaughter in pigs of standard slaughter weight [5, 6] (i.e., 120 kg live weight). The vaccine was very efficient when the recommended protocol was used, with a low incidence of non‐responders [7] (∼ 4.3%). However, there is a knowledge gap regarding physiological responses in other categories of pigs where alternative vaccination protocols are indicated. For example, in production systems with slaughter of older, heavier pigs [8, 9] or rearing entire males (EMs) is not an option due to the higher risk of boar taint [10], especially when pork is intended for high‐quality meat products [11]. Furthermore, immunocastration is of particular interest for young boars culled after a performance test and adult boars from artificial insemination centers [12] for which late vaccination protocols (first vaccination at puberty or at sexual maturity) should be considered.

The timing of the vaccination (and in particular the booster [12]) is an important factor that influences several aspects of production. A late booster is superior with respect to feed conversion rate, economic efficiency, reduced nitrogen excretion [13, 14] and lower environmental impact [15]. Conversely, an early booster effectively reduces aggressive and sexual behavior in males, thus prolonging the period of castration‐like productivity [16] and enhancing meat quality [17]. However, the possibility that hypogonadism is not achieved by immunocastration exists in both alternatives and such pigs are at risk for boar taint. Namely, a higher prevalence of boar taint compounds was reported in piglets immunocastrated early in life [18, 19]; in adult (post‐pubertal) pigs [20]; and pigs of a local breed fattened to higher weight [17]. Despite Leydig cell atrophy [21, 22] and increased Leydig cell nucleus‐to‐cytoplasm ratio [20] (Leydig cells N:C ratio), testes of immunocastrates (IC) remained spermatogenically active 4–6 week after a booster [22]. Although some studies reported longer intervals of non‐recovery [12, 16, 23, 24, 25], perhaps when the interval between a booster and slaughter exceeds 5 week, recovery of Leydig cell function occurs in some boars [22]. This is supported by studies documenting a variable response (morphological level) to immunocastration in young boars, ranging from no response to complete inhibition of spermatogenesis, despite a marked reduction in Leydig cell size [12, 23, 25, 26, 27, 28]. Physiological mechanisms underlying this variability in hypogonadism after immunocastration merit further investigation and could be elucidated using more sensitive approaches like (e.g., quantitative polymerase chain reaction [qPCR] analysis of expression of selected genes in testicular tissues). Some immunocastration‐induced changes in gene expression at the mRNA levels were already identified, including reduced expression of: (i) steroidogenic acute regulatory protein (STAR; [20] responsible for transfer of cholesterol in mitochondria); (ii) enzymes responsible for biosynthesis of steroids from cholesterol, that is, cholesterol side‐chain cleavage enzyme [22], 3‐beta‐hydroxysteroid dehydrogenase [22], hydroxysteroid 17‐beta dehydrogenase 7 (HSD17β7) [20, 22], cytochrome P450 aromatase [22], and 5‐alpha‐reductases [22]; and (iii) insulin‐like peptide 3 (INSL3) and platelet‐derived growth factor receptor alpha (PDGFRα) that are associated with Leydig cell differentiation and functional status [22]. Conversely, upregulation of androgen receptor (AR), follicle‐stimulating hormone receptor (FSHR) and inhibin subunit beta (INHBA), a follicle‐stimulating hormone secretion inhibitor was observed in immunocastrates [20]. Future studies are therefore required to assess effects of alternative immunocastration protocols on gene expression in relation to Leydig cell differentiation and testis functional status. It is hypothesized that this could enable identification of a biomarker for immunocastration in boars.

Therefore, the main objective was to obtain additional insights into physiological effects of androgen deprivation using various intervals between booster and slaughter in boars immunocastrated at the pubertal or post‐pubertal stage and slaughtered at a heavy body weight.

## 2 Methods

### 2.1 Animals and Study Design

All samples used in the study were collected after slaughter. Thus, according to Directive 2010/63/EU [29] and decision of the Ethical Committee of the Veterinary Faculty, University of Ljubljana (decision No. 5‐4‐2018/5), the study was not subject to ethical protocols. The Institute of Preclinical Sciences, Veterinary Faculty of the University of Ljubljana is approved by the Veterinary Administration of the Republic of Slovenia for collection and use of animal by‐products (regulation No 1069/2009) for research purposes (permit No. SI‐B‐07‐22‐07). The use of animal by‐products (i.e., testis samples) for research purposes was also approved by the Veterinary Administration of the Republic of Slovenia (U34453‐1/2024/5).

#### 2.1.1 Main Experiment

**FIGURE 1:**
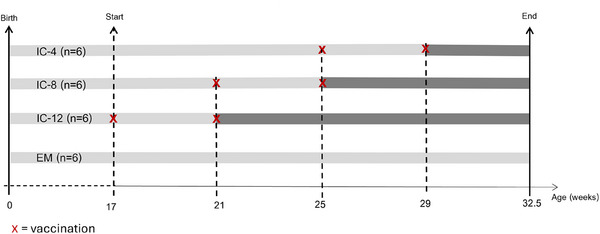
Experimental design with four experimental groups (*n* = 6 per group): entire males (EMs) and three groups of immunocastrates (IC); immunocastrated (Improvac, Zoetis, 2 mL subcutaneous) early, intermediate or late (IC‐12, IC‐8, and IC‐4, respectively), with the booster given 12, 8, or 4 week before slaughter).

To evaluate effects of immunocastration in young male pigs slaughtered at heavy weights, an experiment was conducted on a commercial pig farm using progeny of Large White × Landrace breeds. There were four experimental groups: EM (*n* = 6) and three groups of IC (*n* = 6 per group); immunocastrated by a trained veterinarian according to the manufacturer's instructions (Improvac, Zoetis, 2 mL subcutaneous application) early, intermediate, or late (the booster was done 12, 8, or 4 week, respectively, before slaughter; Figure 1). Boars were housed in group pens according to their treatment group, with ad libitum access to a commercial ration. At the age of 32.5 week and a weight of 152 ± 14 kg, sampling was performed in a commercial abattoir. Animals were slaughtered according to standard procedures (∼ 16 h of feed deprivation, stunning with CO_2_, and immediate vertical bleeding).

#### 2.1.2 Control Experiment

To evaluate outcomes of the main experiment, additional analyses were performed on samples previously collected from young immunocastrated male pigs (YIC) and mature immunocastrated male pigs (MIC), as reported [20].

### 2.2 Measurements and Sampling Procedure

Blood was collected at exsanguination and retained for analysis of antibody titers and testosterone concentrations. Hot carcass weight was recorded, and the genital tract was removed and dissected as described in Fazarinc et al. [7]. Weights of genital tract (the pelvic part of the genital tract, together with the accessory glands and emptied bladder), testes (including epididymides), vesicular gland, and bulbourethral glands (including the urethra) were recorded for each pig. Genital tract, testes, bulbourethral glands, and vesicular glands indices (GTI, TI, BGI, and VGI, respectively) were calculated as respective weight divided by warm carcass weight × 100. Samples of testicular parenchyma were recovered from the area between the tunica albuginea and mediastinum testis of the left testis. Two tissue samples (∼ 1 cm^3^) were collected and fixed in Bouin's solution for histological analysis and a third was stored in RNAlater (Sigma‐Aldrich, Merck KGaA, Darmstadt, Germany) for mRNA expression analysis. The color of the testicular tissue was assessed on a cross‐section of the left testis, according to the International Commission on Illumination (Commission Internationale de l'éclairage [CIE]) CIE L* (lightness), a* (redness), and b* (yellowness) color space in triplicate using a Minolta Chroma Meter CR‐300 (Minolta Co., Ltd., Osaka, Japan) with an 11‐mm aperture, D65 illuminant, calibrated against a white tile. For determination of boar taint compounds, samples of subcutaneous fat were collected from the withers, vacuum packed, and stored at −20°C pending further analysis.

### 2.3 Histological Sample Preparation

After fixation in Bouin's solution for 72 h, testis samples were washed in running tap water, dehydrated, and embedded in paraffin (tissue processor (Leica, Nussloch, Germany) and Tissue‐Tek TEC 5 Tissue Embedding Console System, Sakura Finetek Europe HQ, the Netherlands).

Next, 5‐µm‐thick sections were cut (microtome Leica SM2000R, Nussloch, Germany), stained with hematoxylin and eosin (H&E), mounted and cover slipped (Gemini AS slide stainer and cover slipper ClearVue (Thermo Fisher Scientific, Cheshire, United Kingdom). Images were captured with a Nikon Eclipse Ni‐UM light microscope equipped with a DS‐Fi1 camera and histomorphometric analyses performed using NIS Elements BR 4.6 imaging software (Nikon Instruments Europe B.V., Badhoevedorp, the Netherlands). Areas of Leydig cells and their nuclei were measured, with a minimum of 100 cells analyzed. The Leydig cells N:C ratio was calculated as the nucleus area divided by the cell area, minus the nucleus area.

### 2.4 Chemical Analyses

Percentage of GnRH antibody binding was measured in plasma samples, as described [30]. Briefly, GnRH‐iodination was done using the solid‐phase iodogen method (1 µg iodogen/cup with 200 µCi ^125^I (Na^125^I, Hartmann Analytik GmbH, Braunschweig, I‐RB‐31) and 200 ng GnRH (Fisher Scientific, PEP‐168) diluted in 0.5 M phosphate buffer of pH 7.4). Following a 3‐min incubation, free iodine was separated from the iodinated peptide using an anion‐exchange resin column (specific activity ∼ 200 nCi/ng GnRH). To determine GnRH binding, 15 × 10^3^ cpm ^125^I‐GnRH (corresponding to 17.5 pg GnRH) in 100 µL of 0.1 M phosphate buffer were incubated with 5 µL of plasma and 200 µL of 0.1 M phosphate buffer containing 0.1% BSA at 4°C for 24 h. Subsequently, bound and free separation was done using 0.5% dextran‐coated charcoal in 1 mL H_2_O and centrifugation (1850 × *g*, 20 min). The supernatant was counted for 1 min with a gamma counter and the absolute binding of the biological samples was calculated as counts/total counts. For controls, pooled samples of vaccinated boars with good response (pool+) and non‐vaccinated entire boars (pool−) were measured within each assay. For pool+ and pool−, specific binding was 39% (35%–61%) and 4.4% (1.3%–6.3%), respectively, whereas inter‐assay variations were < 16% and < 23%.

Total testosterone concentrations in plasma samples were determined using a direct in‐house radioimmunoassay kit [30]. Briefly, 20 µL of plasma were incubated with [1,2,6,7‐^3^H]‐testosterone (95.5 Ci/mmol, PerkinElmer, Boston, MA, USA) and antiserum (raised against testosterone‐3‐(O‐carboxymethyl) oxime‐bovine serum albumin [3CMO‐BSA] in a rabbit, used at a final dilution of 1:144,000, 67% cross‐reactivity with 5α‐dihydrotestosterone [5αDHT] and < 2% for other tested steroids). To compensate for substrate effects, charcoal‐treated plasma (20 µL) was added to the calibration curve. Bound/free separation was done (using 0.5 mL ice‐cold solution of 0.5% dextran‐coated charcoal in H_2_O) and subsequent centrifugation (1850 × *g*, 20 min). The supernatant was transferred to counting vials with scintillation fluid and counted using a beta counter. For testosterone, inter‐ and intra‐assay variations were < 6% and < 9%, respectively.

Androstenone concentrations were measured in the collected adipose tissue samples by high‐performance liquid chromatography (HPLC), as described [31]. Briefly, adipose tissue samples were liquefied in a microwave oven for 3 × 1 min at 350 W, transferred to 2.5 mL tubes, and centrifuged for 20 min at 11,200 × *g* and 20°C. After centrifugation, 0.5 ± 0.01 g water‐free liquid fat was transferred to 2.5 mL tubes and 1 mL of methanol containing internal standard (0.496 mg/L androstenone) was added to each tube. After stirring for 30 s, tubes were incubated in an ultrasonic water bath for 5 min at 30°C, placed on ice for 20 min, and centrifuged for 20 min at 11,200 × *g* at 4°C. For androstenone determination, 50 µL of the supernatant was subjected to derivatization with dansylhydrazine for exactly 2 min. A 10‐µL aliquot of the derived mixture was injected into an HPLC column for detection of fluorescence (excitation at 346 nm and emission at 521 nm) on an HP1200 (Agilent Technologies, Waldbronn, Germany). Concentrations were expressed per gram of liquid fat, the detection limit of the method was 0.24 µg/g, and inter‐ and intra‐assay variations were < 8% and < 10%, respectively.

### 2.5 RNA Extraction, cDNA Synthesis, and Quantitative Polymerase Chain Reaction

Total RNA was extracted from testicular tissue samples using an RNeasy Mini Kit (Qiagen, Hilden, Germany; catalog number: 74104). The extraction protocol included an on‐column DNase digestion step with RNase‐Free DNase Set (Qiagen). The 260/280 and 260/230 absorbance ratios were determined using a UV–VIS Lambda 25 spectrophotometer (Perkin Elmer, Waltham, MA, USA) to check the purity of the extracted RNA samples. Integrity and quality of the RNA samples were controlled by Qubit RNA IQ assay using Invitrogen Qubit 4 Fluorometer. cDNA was synthesized using a High‐Capacity cDNA Reverse Transcription Kit (Thermo Scientific GmbH, Vienna, Austria; catalog number: 4368814) according to the manufacturer's instructions. First‐strand cDNA synthesis was performed with reverse transcription (RT) random primers and reverse transcriptase (Thermo Scientific GmbH) using 1.5 µg of each RNA sample, with 260/280 and 260/230 ratios close to 2.0 and the RNA integrity number ≥ 8.

A QuantStudio 5 Real‐Time PCR System (Applied Biosystems, Thermo Scientific GmbH) was used for qPCR, as described in refs. [20, 32]. Primers and fluorescent 6‐FAM dye‐labeled minor‐groove‐binder probes/predesigned assays (Table 1) were from Applied Biosystems (Thermo Scientific GmbH). Beta‐2‐microglobulin (B‐2‐M) and eukaryotic ribosomal (r) 18s RNA (18s rRNA) were used as endogenous controls for normalization. Quantitative PCRs were performed in a final volume of 10 µL containing 4.5 µL of each RT sample (diluted 10‐fold), 5 µL of TaqMan Universal Master Mix II, and 0.5 µL of TaqMan Gene Expression Assay under the following conditions: one cycle of 50°C for 2 min and one cycle of 95°C for 10 min, followed by 45 cycles of 15 s at 95°C and 1 min at 60°C. Each reaction was performed in triplicate. Results were calculated from the threshold cycle (Ct) that was fixed at 0.10. A Ct value > 35 was regarded as the cutoff. The Ct values for B‐2‐M and 18s RNA were used as controls to normalize Ct values [33, 34] of evaluated preselected target transcripts (Table 1). Delta Ct (ΔCt) values were calculated using the comparative Ct method (ΔCt = Ct target transcript − Ct geometric mean of controls). Relative changes in expression of the examined target transcripts (fold change, FC) were determined using the 2^−ΔΔCt^ method [35]. The PCR efficiency (> 90%) of the analyzed genes was derived from standard curves consisting of four 10‐fold dilutions of cDNAs performed on pooled samples. Relative Quantification Analysis Module, version 3.9 (Thermo Fisher Scientific, Applied Biosystems) was used for data analysis.

**TABLE 1:** List of predesigned TaqMan gene expression assays used for quantitative PCR.

Full gene name	Gene	Amplicon length	Assay ID	Function/use
Estrogen receptor 1	ESR1	70	Ss03383398_u1	Mediates estrogenic effects
Estrogen receptor 2	ESR2	84	Ss03391479_m1	Mediates estrogenic effects
Follicle‐stimulating hormone receptor	FSHR	99	Ss03384581_u1	Control of gonadal function and reproduction
Luteinizing hormone/choriogonadotropin receptor	LHCGR	64	Ss03384991_u1	Control of gonadal function and reproduction
Inhibin subunit beta A	INHIBA	90	Ss03393536_s1	Forms a pituitary FSH secretion inhibitor
Inhibin subunit alpha	INHA	76	Ss03383260_u1	Forms a pituitary FSH secretion inhibitor
Gonadotropin‐releasing hormone receptor II	GNRHR‐II	63	Ss03391559_m1	Control of gonadal function and reproduction
Androgen receptor	AR	86	Ss03822350_s1	Mediates androgenic effects
Steroidogenic acute regulatory protein	STAR	73	Ss03381250_u1	Transfer of cholesterol into the mitochondria
Hydroxysteroid 17‐beta dehydrogenase 7	HSD17β7	61	Ss04246893_m1	Biosynthesis of steroid hormones from cholesterol
Betaine‐homocyteine S‐methyltransferase	BHMT	83	Ss03374598_m1	Adult Leydig cell marker
Insulin‐like peptide 3	INSL3	139	Ss03393127_u1	Marker of Leydig cell function
Corticotropin‐releasing hormone receptor 1	CRHR1	65	Ss03373289_g1	Fetal Leydig cell marker
Platelet‐derived growth factor receptor alpha	PDGFRα	59	Ss04955107_g1	Stem Leydig cell marker
Beta‐2‐microglobulin	B‐2‐M	60	Ss03391154_m1	Endogenous control
Eukaryotic ribosomal (r) 18S rRNA	18S rRNA	69	Hs03003631_g1	Endogenous control

### 2.6 Statistical Analyses

**FIGURE 2:**
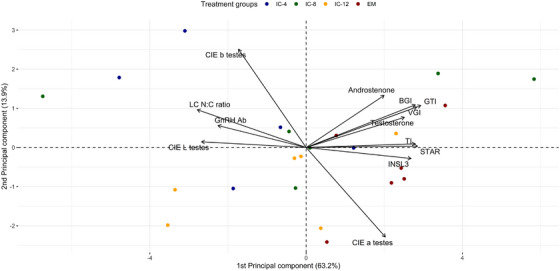
Principal component analysis (PCA) biplot showing the distribution of entire male (EM) pigs (red dots, *n* = 6) and immunocastrated male pigs (IC; *n* = 17, 2 vaccinations with Improvac, 2 mL subcutaneous application) boostered 4 (IC‐4; blue dots), 8 (IC‐8; green dots), or 12 (IC‐12; yellow dots) weeks prior to slaughter. The first two principal components of PCA explain 77.1% of the total variation. Variables strongly correlated with PC1 are indicated by arrows; those that are closer to each other are positively correlated and those that are in opposite directions are negatively correlated. Row data were used for the analysis. Selected variables (*n* = 13): Androstenone, androstenone concentration in backfat (µg/g liquid fat); Testosterone, testosterone concentration at slaughter (ng/mL plasma); GnRH Ab, gonadotropin‐releasing hormone antibody binding at slaughter (%); GTI, genital tract index, calculated as the genital tract weight (weight of the pelvic part of the genital tract, accessory glands, and emptied bladder) divided by warm carcass weight × 100; TI, testis index, calculated as testis weight (weight of both testes, with epididymides included) divided by warm carcass weight × 100; BGI, bulbourethral gland index, calculated as bulbourethral gland weight (weight of both bulbourethral glands and urethra) divided by warm carcass weight × 100; VGI, vesicular gland index, calculated as vesicular gland weight divided by warm carcass weight × 100; LC N:C ratio, Leydig cells nucleus‐to‐cytoplasm ratio, calculated as nucleus area divided by cytoplasm area; CIE L testes, lightness; CIE a testes, redness and CIE b testes, yellowness of testicular parenchyma cross section; STAR, delta cycle threshold (ΔCt) of steroidogenic acute regulatory protein; INSL3, ΔCt of Insulin‐like peptide 3.

**FIGURE 3:**
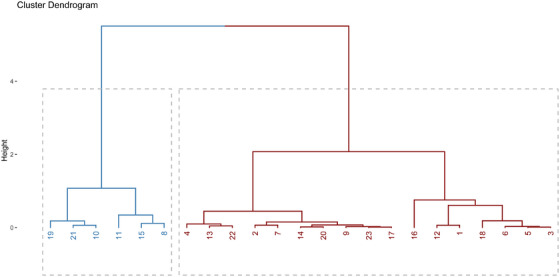
The cluster dendrogram of individual pigs [1–6: entire male (EM) pigs, 7–11: immunocastrated male pigs (IC) boostered 4 week before slaughter (IC‐4), 12–17: IC boostered 8 week (IC‐8) before slaughter; 18–23: IC boostered 12 week before slaughter (IC‐12)] generated by hierarchical clustering on principal component analysis (cf. Figure 2) of selected variables (*n* = 13) related to sexual development and responses to immunocastration. Note that cluster dendrogram tree is divided into two parts (clusters)—**blue**, individuals with ID 8, 10, 11, 15, 19, 21: characterized by plasma testosterone < 1 ng/mL and thus subsequently referred as IC‐LT; and a **red** part, individuals with ID 1–6: entire males (EMs) and individuals with ID 7, 9, 12–14, 16–18, 20, 22, and 23 a part of IC clustered with EM, characterized by plasma testosterone > 1 ng/mL and thus subsequently referred as IC‐HT.

All statistical analyses were performed using R statistical software (Version 4.4.1). Individual pigs were considered an experimental unit. For fat androstenone concentrations, detection limits (0.24 µg/g liquid fat) were assigned to individuals below this limit. The principal component analysis (PCA) was performed on raw, anonymized data identified by animal ID numbers, without reference to sex or treatment group using the R package FactoMineR [36]. Thirteen investigated variables related to sexual development and response to immunocastration were included: GTI; TI; BGI; VGI; CIE L*, a*, b* color parameters of testicular parenchyma; plasma testosterone and fat androstenone concentrations; Leydig cells N:C ratio, plasma GnRH antibody titer; the normalized expression level (ΔCt) of STAR; the expression level (ΔCt) of INSL3, followed by a clustering procedure to evaluate effects of immunocastration, using the statistical approach as described [20]. The first two PCA components had eigenvalues > 1 and together accounted for 77.1% of the inertia. The PCA biplot of individual animals from all experimental groups distinguished by color and variables related to sexual development and response to immunocastration is shown in Figure 2. Subsequent hierarchical clustering based on the dendrogram shape and the bar graph of the inertial gain [36] resulted in a cluster dendrogram tree (Figure 3) with clear division into two parts (clusters, i.e., blue and red). Blue part comprised individual IC animals (*n* = 6) characterized by plasma testosterone concentrations < 1 ng/mL and was therefore labeled as IC‐LT group. The remaining IC (*n* = 11) clustered with EM (red cluster) and was considered as separate study group. This group of IC had plasma testosterone > 1 ng/mL and was therefore labeled “IC‐HT.”

Correlation analysis was performed using the R package Hmisc [37], comparing variables related to sexual development and immunocastration responses (GTI; TI; BGI; VGI; CIE L* color parameter of testicular parenchyma; plasma testosterone and fat androstenone concentrations; Leydig cells N:C ratio, plasma GnRH antibody titer) with normalized mRNA expression levels (ΔCt) of selected genes in testicular parenchyma (Estrogen receptor 2 [ESR2], FSHR, luteinizing hormone/choriogonadotropin receptor [LHCGH], AR, STAR, HSD17β7, INSL3, and PDGFRα).

A non‐parametric statistical model (pairwise Wilcoxon rank‐sum test with Bonferroni adjustment) was used to evaluate differences among the four studied groups (IC‐4, IC‐8, IC‐12, and EM) for variables related to sexual development and data presented as medians with interquartile ranges. Student's *t*‐test was used for statistical analysis of relative testicular mRNA expressions. The following comparisons were made IC‐LT versus EM, IC‐HT versus EM, and IC‐LT versus IC‐HT and data presented as mean FC. FC > 1.5 and *p* < 0.05 were set for significant change in relative expression.

## 3 Results

### 3.1 Reproductive Organs, GnRH Antibody Titer, Hormones and Boar Taint Compounds of Young Immunocastrated and Entire Male Pigs, Slaughtered at Heavy Weight

As expected, at slaughter, IC had a higher GnRH antibody titer than EM (*p* < 0.004; Table 2). Testosterone concentrations were lower in IC‐4 compared with EM, and intermediate in IC‐8 and IC‐12 (*p* = 0.041, Table 2). Backfat androstenone concentrations were highly variable (median values ranging from 0.24 in IC‐4 to 2.4 µg/g liquid fat in EM) and tended to be lower in IC‐4 compared with EM control (*p* = 0.067).

**TABLE 2:** Effects of gonadotropin‐releasing hormone 1 suppression timing on sexual development and responses to immunocastration (*n* = 23).

End point	IC‐4^a^ (*n* = 5)	IC‐8^a^ (*n* = 6)	IC‐12^a^ (*n* = 6)	EM (*n* = 6)	*p* value^b^
Genital tract index^c^ (%)	0.54 [0.13]	0.61 [0.21]	0.49 [0.23]	0.63 [0.13]	0.276
Testis index^d^ (%)	0.79 [0.28]	0.69 [0.33]	0.68 [0.42]	0.84 [0.10]	0.452
Vesicular gland index^e^ (%)	0.25 [0.12]	0.21 [0.07]	0.18 [0.13]	0.29 [0.12]	0.376
Bulbourethral glands index^f^ (%)	0.12 [0.02]	0.19 [0.12]	0.14 [0.05]	0.21 [0.04]	0.242
Color of testicular tissue CIE^g^ L	55.6 [2.9]	55.2 [4.6]	55.7 [5.6]	51.1 [3.4]	0.144
Color of testicular tissue CIE^g^ a	14.2 [3.4]	16.2 [1.4]	16.7 [1.4]	17.8 [1.8]	0.077
Color of testicular tissue CIE^g^ b	9.0[3.4]	8.3 [1.2]	7.5 [1.6]	6.8 [1.2]	0.102
Leydig cells N:C ratio^h^	0.30^b^ [0.22]	0.21^ab^ [0.05]	0.24^ab^ [0.10]	0.21^a^ [0.04]	0.033
GnRH antibody binding (%)	46.1^b^ [16.3]	29.1^b^ [18.8]	41.7^b^ [3.6]	7.9^a^ [0.04]	0.004
Plasma testosterone (ng/mL)	0.65^a^ [4.8]	8.14^ab^ [13.8]	6.70^ab^ [4.3]	10.5^b^ [5.2]	0.041
Fat androstenone (µg/g liquid fat)	0.24 [0.36]	0.72 [5.1]	0.55 [0.8]	2.4 [1.8]	0.086

Abbreviations: CIE, Commission Internationale de l'Elcairage; EM, entire male pigs; IC, immunocastrated male pigs.

^a^ Vaccination with Improvac (2 mL, s.c. application, Zoetis using various intervals between booster and slaughter: 4, 8, or 12 week (IC‐4, IC‐8, and IC‐12, respectively).

^b^ Wilcoxon rank‐sum test with Bonferroni adjustment.

^c^ Calculated as the genital tract weight (weight of the pelvic part of the genital tract, accessory glands, and emptied bladder) divided by warm carcass weight × 100.

^d^ Calculated as the testis weight (weight of both testes, with epididymides included) divided by warm carcass weight × 100.

^e^ Calculated as the vesicular gland weight divided by warm carcass weight × 100.

^f^ Calculated as bulbourethral gland weight (weight of both bulbourethral glands and urethra) divided by warm carcass weight × 100.

^g^ CIE L, a, b color space; L = lightness, higher number denotes a lighter color; a = redness, higher number denotes redder; b = yellowness, higher number denotes yellower.

^h^ Calculated as the Leydig cell's nucleus area divided by the cytoplasm area.

Differences in GTI, TI, BGI and VGI were not significant (*p* > 0.10, Table 2). The testicular parenchyma of IC‐4 tended to be less red (*p* = 0.077, Table 2) compared with EM, whereas no difference was observed for lightness or yellowness (*p* > 0.10, Table 2). Leydig cells N:C ratios were lower in EM compared with IC‐4 and intermediate in IC‐8 and IC‐12 (*p* = 0.033, Table 2).

### 3.2 Testicular Histology

**FIGURE 4:**
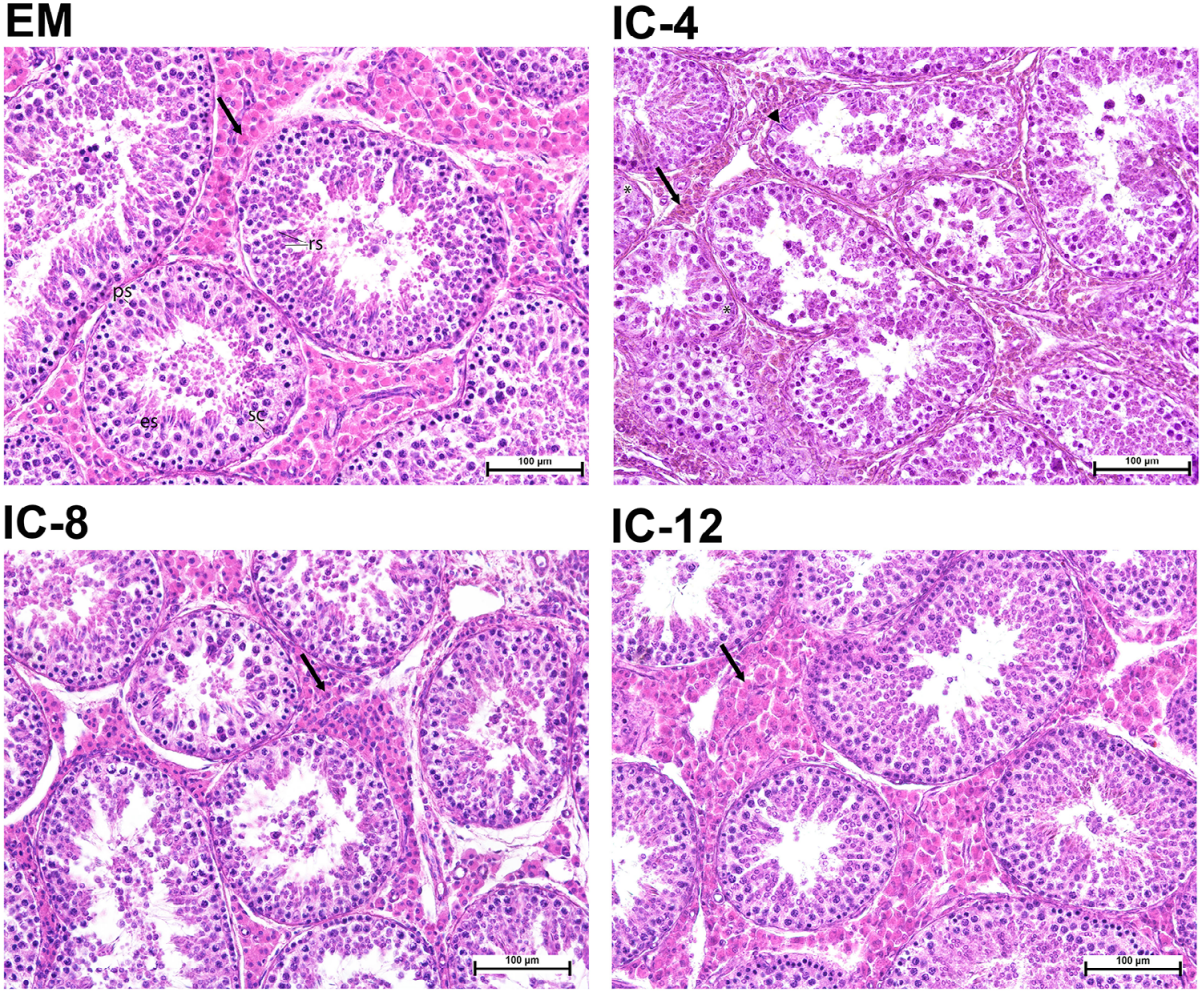
Representative photomicrographs (objective 40×) of testis cross‐sections; note the seminiferous tubules and interstitial Leydig cells of immunocastrated male pigs (IC) compared with entire male (EM) pigs. IC were immunocastrated (vaccination and booster with Improvac, 2 mL subcutaneous) using various intervals between booster and slaughter: 4, 8, or 12 week (IC‐4, IC‐8, and IC‐12, respectively). Note the drastic reduction in the number and size of Leydig cells (black arrows) in the IC‐4 (right top photomicrograph). Spermatid heads retention deep within the seminiferous epithelium (black arrowheads) and signs of seminiferous epithelium vacuolation (asterisks) are present in IC‐4. Hematoxylin and eosin staining, scale bar = 100 µm; es, elongated spermatids; ps, primary spermatocytes; rs, round (spherical) spermatids; sc, Sertoli cell.

Representative photomicrographs of H&E‐stained testis cross‐sections for each experimental group are shown (Figure 4). The EM control (upper left micrograph in Figure 4) had a normal EM testicular parenchyma; seminiferous tubules with active spermatogenesis and a large interstitial compartment predominantly occupied by Leydig cells. In contrast, the IC‐4 (upper right micrograph in Figure 4) had histological changes suggestive of androgen deficiency, including degeneration of spermatocytes and spermatids close to the basal compartment of the seminiferous epithelium, disintegration of the germinal epithelium with varying degrees of degeneration, vacuolization, and marked Leydig cell atrophy; the latter was indicated by a significantly increased Leydig cells N:C ratio (cf. Table 2) and a substantially decreased plasma testosterone concentration. Leydig cell atrophy was less evident in groups IC‐8 (lower left micrograph in Figure 4) and IC‐12 (lower right micrograph in Figure 4), corroborated by Leydig cell morphometry and plasma testosterone data (cf. Table 2).

### 3.3 Testicular mRNA Expression of Genes Related to Testicular Function and Steroidogenesis

Comparison of relative testicular mRNA expression of selected genes related to the control of testicular function and steroidogenesis is presented based on hierarchical clustering for pigs with different responses to immunocastration (cf. Figure 3; groups IC‐HT and IC‐LT) compared with EM and for IC‐LT versus IC‐HT (Table 3). Expression levels of FSHR and ESR2 were higher in the IC‐LT compared with the IC‐HT cluster (FC > 1.5 and *p* < 0.05). Conversely, expression levels of LHCGH, STAR and INSL3 were lower in the IC‐LT than in the IC‐HT and EM (FC > 1.5 and *p* < 0.05). ESR1 and HSD17β7 mRNA expression levels were significantly lower in both IC‐HT and IC‐LT clusters compared with EM (FC > 1.5 and *p* < 0.05), whereas expression of PDGFRα tended to be higher in IC‐LT than in IC‐HT and lower (at the cutoff for FC) in IC‐HT than in EM. Conversely, the expression levels of FSHR and ESR2 were significantly higher in the IC‐LT cluster compared with the IC‐HT cluster (FC > 1.5 and *p* < 0.05).

**TABLE 3:** Comparison of gene expression related to testicular function, steroid metabolism, and Leydig cell functional/differentiation status in testicular tissue of immunocastrated male pigs (IC^a^) with different responses to immunocastration compared with entire male (EM) pigs and between IC with different responses to immunocastration.

	Fold change (FC)^b^					
Target transcript	IC‐HT vs. EM	*p* value	IC‐LT vs. EM	*p* value	IC‐LT vs. IC‐HT	*p* value
ESR1	−1.69	0.034	−1.83	0.048	−1.08	0.788
ESR2	−1.09	0.715	1.69	0.065	1.84	0.024
FSHR	−1.76	0.106	1.57	0.176	2.77	< 0.001
LHCGR	−1.43	0.183	−3.72	0.002	−2.61	0.007
GNRHR‐II	−1.34	0.121	−1.04	0.845	1.29	0.045
INHBA	−1.29	0.204	1.06	0.769	1.37	0.076
INHA	1.08	0.570	1.04	0.855	−1.05	0.816
AR	−1.34	0.160	1.00	0.973	1.34	0.092
STAR	−1.44	0.494	−57.0	< 0.001	−39.5	0.002
HSD17β7	−1.55	0.024	−1.89	0.004	−1.22	0.280
BHMT	−1.12	0.462	−1.30	0.081	−1.17	0.215
INSL3	1.09	0.700	−12.0	0.003	−13.0	0.003
CRHR1	−1.48	0.389	−1.73	0.079	−1.16	0.733
PDGFRα	−1.49	0.027	−1.11	0.499	1.34	0.073

Abbreviations: AR, androgen receptor; BHMT, betaine‐homocyteine S‐methyltransferase; CRHR1, corticotropin‐releasing hormone receptor 1; ESR1, estrogen receptor 1; ESR2, estrogen receptor 2; FSHR, follicle‐stimulating hormone receptor; GnRHR‐II, gonadotropin‐releasing hormone receptor‐II; HSD17β7, hydroxysteroid 17‐beta dehydrogenase 7; INHA, inhibin subunit alpha; INHBA, inhibin subunit beta A; INSL3, insulin‐like peptide 3; LHCGH, luteinizing hormone/choriogonadotropin receptor; PDGFRα, platelet‐derived growth factor receptor alpha; STAR, steroidogenic acute regulatory protein.

^a^ Immunocastrated male pigs (IC; vaccination and booster with Improvac, 2 mL subcutaneous) using three intervals (4, 8, or 12 week) between booster and slaughter divided as the one clustered with EM according to principal component analysis followed by hierarchical clustering (cf. Figure 3; characterized by plasma testosterone > 1 ng/mL and thus labeled as IC‐HT) and the separate cluster of IC (with plasma testosterone < 1 ng/mL; labeled IC‐LT); EM, entire (uncastrated) male pigs.

^b^ The mean fold changes (FCs) in the expression were calculated using the 2^−ΔΔCt^ method as described in Material and methods. FC values < 1 were substituted with a negative inverse of the original FC values. FC cutoff of 1.5 and *p* < 0.05 was set for significant differential expressions.

### 3.4 Testicular mRNA Expression of Genes Related to Leydig Cell Differentiation and Functional Status

To further validate potential effects of immunocastration on Leydig cell markers of differentiation and functional status (i.e., BHMT, INSL3, CRHR1, and PDGFRα), cDNA from testes recovered in previous studies [20] were additionally analyzed and results summarized in Table 4. These pigs were subjected to the two‐dose vaccination schedule recommended by the manufacturer, with a minimum interval of 4 weeks between V1 and V2 and 4 weeks between V2 and slaughter, namely, young IC (YIC), young EM (YEM), and mature IC (MIC), clustered as YEM, MIC with plasma testosterone > 1 ng/mL (MIC‐HT), YIC with plasma testosterone > 1 ng/mL (YIC‐HT), MIC with plasma testosterone < 1 ng/mL (MIC‐LT), and YIC with plasma testosterone < 1 ng/mL (YIC‐LT). Regardless of age at V1, the expression of INSL3 was significantly decreased in both groups of ICs with plasma testosterone < 1 ng/mL (YIC‐LT and MIC‐LT) compared with EM and IC with plasma testosterone > 1 ng/mL (YIC‐HT and MIC‐HT). Validation on additional samples also showed that hinted change in PDGFRα expression was only confirmed in YIC (1.63‐fold higher in YIC‐LT than in YIC‐HT).

**TABLE 4:** Comparison of gene expression related to Leydig cell differentiation/functional status in testicular tissue of mature (MIC) and young (YIC) immunocastrated male pigs (IC) (vaccination and booster with Improvac, 2 mL subcutaneous) with different responses to immunocastration compared with entire male (EM) pigs.

	Fold change^a^ *(p* value)			
Compared groups	BHMT	INSL3	CRHR1	PDGFRα
YIC‐HT vs. YEM	1.02 (0.894)	1.08 (0.680)	−1.39 (0.448)	−1.33 (0.066)
YIC‐LT vs. YEM	1.01 (0.997)	−15.3 (< 0.001)	−1.2 (0.438)	1.23 (0.193)
MIC‐HT vs. YEM	−1.16 (0.305)	1.42 (0.191)	−1.19 (0.373)	1.09 (0.309)
MIC‐LT vs. EM	−1.49 (0.134)	−2.77 (0.002)	−2.28 (0.538)	1.01 (0.336)
YIC‐LT vs. YIC‐HT	−1.00 (0.932)	−16.6 (< 0.001)	1.16 (0.762)	1.63 (0.019)
MIC‐LT vs. MIC‐HT	−1.29 (0.373)	−3.93 (< 0.001)	−1.91 (0.909)	−1.07 (0.956)

Abbreviations: BHMT, betaine‐homocyteine S‐methyltransferase; CRHR1, corticotropin‐releasing hormone receptor 1; INSL3, insulin‐like peptide 3; MIC, mature immunocastrated male pig; MIC‐HT, MIC with plasma testosterone > 1 ng/mL; MIC‐LT, MIC with plasma testosterone < 1 ng/mL; PDGFRα, platelet‐derived growth factor receptor alpha; YEM, young entire (uncastrated) male pigs; YIC, young immunocastrated male pigs; YIC‐HT, YIC with plasma testosterone > 1 ng/mL; YIC‐LT, YIC with plasma testosterone < 1 ng/mL.

^a^ The mean fold changes (FCs) in the expression were calculated using the 2^−ΔΔCt^ method, as described in Material and methods. FC values < 1 were substituted with a negative inverse of the original FC values. FC cutoff of 1.5 and *p* < 0.05 was set for significant differential expressions.

### 3.5 Correlations Between Investigated Variables Related to Immunocastration Response and Normalized Expression (ΔCt) of Selected Genes in Testicular Tissue

**FIGURE 5:**
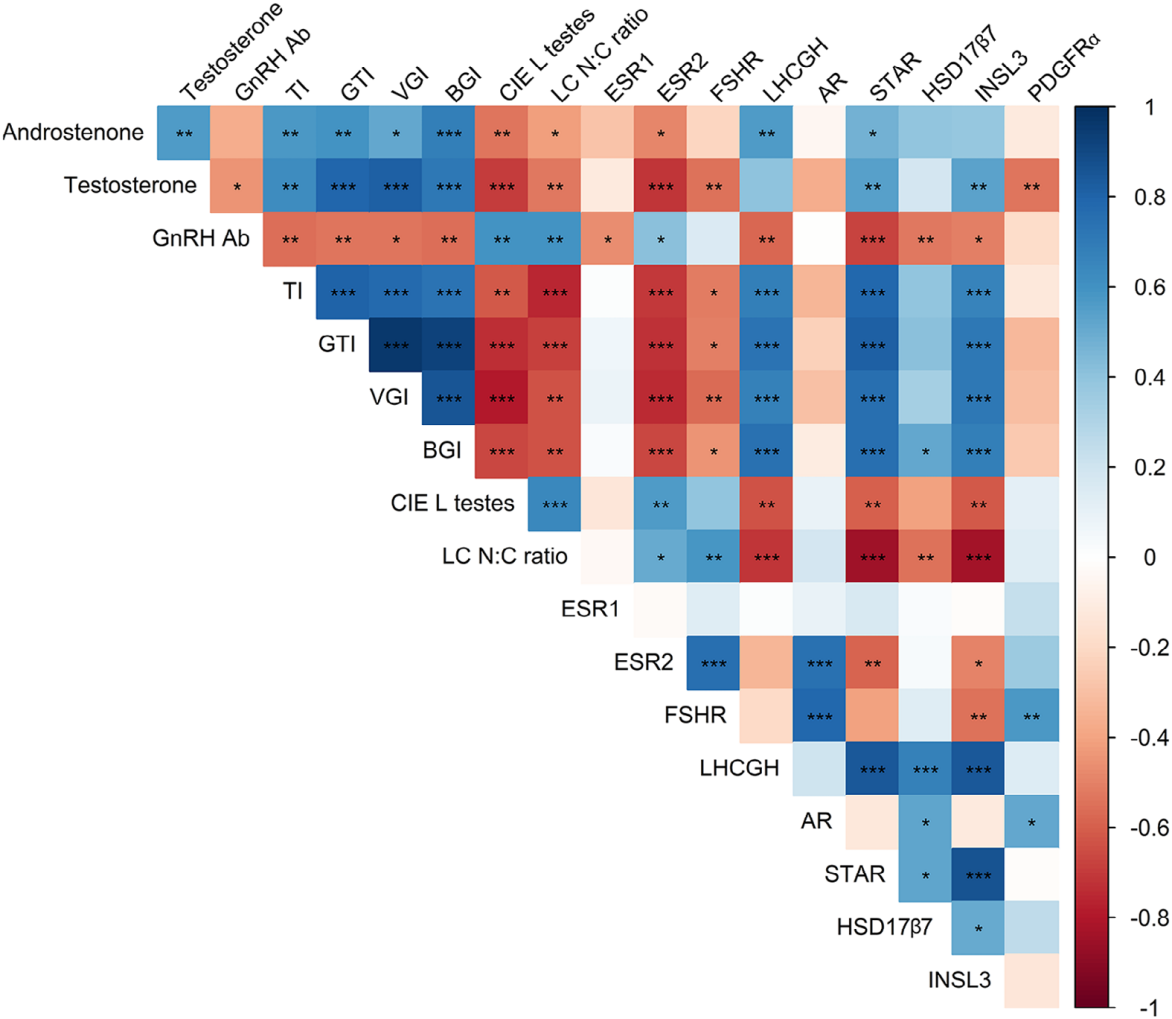
Correlation matrix of variables associated with reproductive function, response to immunocastration and normalized expression (ΔCt) of selected genes in testicular parenchyma. Significant (*p* > 0.05) Pearson`s correlations between variables are displayed by color: positive correlations with blue, and negative with red (asterisks denote significant correlation; *p < 0.05, **p < 0.01, ***p < 0.001). Androstenone, backfat androstenone concentration; AR, androgen receptor; BGI, bulbourethral gland index; CIE L testes, lightness of testicular parenchyma cross section; ESR1, estrogen receptor 1; ESR2, estrogen receptor 2; FSHR, follicle‐stimulating hormone receptor; GnRH Ab, plasma GnRH antibody titer at slaughter; GTI, genital tract index; HSD17β7, hydroxysteroid 17‐beta dehydrogenase 7; INSL3, insulin‐like peptide 3; LC N:C ratio, Leydig cells nucleus‐to‐cytoplasm ratio; LHCGH, luteinizing hormone/choriogonadotropin receptor; PDGFRα, platelet‐derived growth factor receptor alpha; STAR, steroidogenic acute regulatory protein; Testosterone, blood testosterone concentration at slaughter; TI, testes index; VGI, vesicular gland index.

Pearson's correlations between investigated variables related to sexual development, responses to immunocastration and normalized (ΔCt) expression of selected target genes associated with the control of testicular function and steroidogenesis in testicular tissue are shown in Figure 5. Correlations between anatomical (GTI, TI, VGI, and BGI) and histomorphometrical parameters (lightness of testicular tissue and Leydig cells N:C ratio), GnRH antibody binding, plasma testosterone concentration, and fat androstenone concentrations were as reported [7, 20]. Among the selected genes tested, expression of INSL3, STAR, and LHCGH had strong correlations with most studied variables. INSL3 was positively associated with testosterone, anatomical parameters (GTI, TI, VGI, BGI), and expression of STAR, LHCGH, and HSD17β7, but negatively with GnRH binding, testis lightness, Leydig cell N:C ratio, ESR2, and FSHR. STAR expression mirrored INSL3, with positive correlations with steroid concentrations and testis indices, and negative to ESR2, GnRH binding, and Leydig cell N:C ratio. LHCGH correlated positively with INSL3, STAR, and testosterone concentrations, and negatively with GnRH binding and Leydig cell N:C ratio. Fewer correlations were noted for PDGFRα (positive with AR and FSHR, negative with testosterone), AR (positive with ESR2, FSHR, HSD17β7, PDGFRα), and ESR2 (negatively linked to INSL3, STAR, and testis traits).

## 4 Discussion

Increased GnRH antibody titers confirmed that all IC had an immunological response to vaccination, regardless of vaccination protocol. However, responses at the testicular level were variable, with great individual differences in the genital tract, testes, and accessory sex glands indexes, as well as the color of testicular parenchyma. This may be due to the late timing of the first vaccinations (17, 21, and 25 week of age) and boosters (21, 25, and 29 week of age) in all alternative protocols tested, which did not interfere with critical reproductive tract development periods in boars, including a brief neonatal exposure to elevated estrogens and androgens, the juvenile phase with low sex steroids (6–11 week), and the subsequent rise in estrogens and androgens until 16 week [38, 39]. High inter‐animal variability in regression of testicular function following immunocastration, which increased as the delay between GnRH suppression and evaluation was prolonged, has been reported in several studies [25, 26, 27, 40, 41]. The critical antibody level to suppress testicular steroid production is still undefined. In a recent study evaluating effectiveness of anti‐GnRH vaccination on 282 boars [7], the antibody titer required to diminish Leydig cell function differed between successfully and unsuccessfully immunized individuals within a narrow range (45.5% vs. 38.7% GnRH antibody binding), resulting in higher plasma testosterone (0.48 vs. 3.2 ng/mL) and fat androstenone (0.24 vs. 0.95 µg/g fat). In the present study, the Leydig cells N:C ratio, an indicator of Leydig cell activity, was 1.4‐fold higher in IC‐4 compared with EM control, whereas in IC‐8 and IC‐12, the Leydig cells N:C ratio was intermediate, indicating possible recovery of Leydig cells endocrine activity. Accordingly, plasma testosterone concentrations were 16.5‐fold lower in IC‐4 compared with EM controls, and intermediate in IC‐8 ‐and IC‐12, in which they greatly exceeded the threshold (0.5 ng/mL) for resumption of testicular function [27] and were comparable to values reported for EM (0.17–50 ng/mL [42, 43, 44]). Similarly, Wicks et al. [40] reported that serum testosterone concentrations began to increase ∼ 8 week after immunocastration with Improvac. Considering the delay between the recovery of testosterone at the systemic level and production at the testicular level, recovery of Leydig cells function is likely to occur earlier than 8 week after booster [27]. When a two‐dose vaccination regimen was used in sexually mature animals, the interval for return to Leydig cell function is likely shorter [20]. Considering that the peak of puberty is ∼ 22 week [45], the booster was administered at the pubertal or early post‐pubertal transition stage in the present study (at 21, 25, or 29 week of age), resulting in variable responses. Namely, in individual IC, recovery of Leydig cells and some developing spermatids were observed histologically, regardless of vaccination protocol. However, changes were more pronounced with a prolonged delay between immunocastration and slaughter (groups IC‐8 and IC‐12; Figure 4). The PCA with hierarchical clustering further supported this observation, as IC with signs of Leydig cells recovery clustered together with EM (red part of cluster dendrogram, Figure 3). In agreement with Fazarinc et al. [7], IC clustered with EM were characterized by lower GnRH antibody binding percentage (32.9% vs. 47.1%; Supporting Information Table 1), higher serum testosterone (7.04 vs. 0.56 ng/mL; Supporting Information Table 1), higher fat androstenone (0.82 vs. 0.24 µg/mL liquid fat; Supporting Information Table 1), higher GTI (0.58 vs. 0.29; Supporting Information Table 1), TI (0.79 vs. 0.37; Supporting Information Table 1), VGI (0.22 vs. 0.05; Supporting Information Table 1), BGI (0.16 vs. 0.09; Supporting Information Table 1), and darker testis parenchyma (53.9 vs. 57.3 CIE L* color parameter). Additionally, there was a lower Leydig cells N:C ratio (0.22 vs. 0.42; Supporting Information Table 1). In addition to testosterone and Leydig cells N:C ratio, GTI, TI, VGI, and BGI were good indicators of successful androgen deprivation and correlated well with GnRH antibody titer and fat androstenone concentrations, consistent with previous results proposing genital tract or testis weight as reliable indicators for discriminating carcasses with high versus low boar taint [7, 20, 46]. Combining current and past results, threshold values for successful immunocastration could be suggested. Namely, GnRH antibody percentage > 40%, plasma testosterone < 1 ng/mL, GTI < 0.45, TI < 0.55, and/or Leydig cells N:C ratio > 0.30 are indicative of androgen deficiency, with low androsterone concentrations (≤ 0.24 ng/mL liquid fat).

However, all above indicators require additional sampling, dissection, measurement, and/or analysis. Therefore, there is a desire for identifying an immunocastration biomarker in pigs that could be used for either live animals or carcasses. Some research has already been conducted, focusing on differences in mRNA expression in testicular tissue between boars and IC [20, 22]. In the present study, lower expression levels of STAR, responsible for the transfer of cholesterol from the outer to the inner mitochondrial membrane [47], together with lower expression level of HSD17β7, responsible for biosynthesis of steroids from cholesterol and the reduction of estrone [48], and substantially lower expression level of LHCGR (Table 3) responsible for regulation of steroidogenesis was present in IC‐LT group. This was consistent with decreased testosterone production and regression of Leydig cells (increase in Leydig cells N:C ratio) observed after immunocastration and agreed with previous findings [20, 22]. The higher FSHR expression in IC‐LT group, localized on Sertoli cells [49], supported the absence of androgen negative feedback [20]. Because GnRH vaccination reduced expression of LHCGR, primarily expressed by Leydig cells, leading to short‐term refractoriness of Leydig cells upon LH begin to rise [27]. Perhaps differences in LHCGR expression have a key role in the variability of immunocastration efficacy observed in this and previous studies on alternative vaccination protocols and are linked to individual differences in restoration of Leydig cell function. No previous study had directly linked immunocastration to alterations in ESR1/ESR2 expression (FC < 2; Table 3); nonetheless, decreased ESR1 expression in ICs compared with EMs was consistent with results of an association study highlighting the role of ESR1 in promoting spermatogenesis and maintaining testicular function in boars [50].

Additionally, Pawlicki et al. [22] reported moderate decrease in PDGFRα protein level in YIC and suggested that immunocastration affected Leydig cell development and the exchange of fetal, perinatal and adult Leydig cell populations. The observed difference in PDGFRα mRNA expression between pigs with different responses to immunocastration (higher in YIC‐LT than in YIC‐HT; Table 4) implied increased proliferation and differentiation of stem Leydig cells prior to restoration of Leydig cell function. Notably, this difference was absent in mature ICs (comparison MIC‐LT vs. MIC‐HT; average age at slaughter 68.6 week [20]), that might suggest an age‐dependent decline in stem Leydig cell number/responsiveness. PDGFRα transcript level also correlated negatively with plasma testosterone concentration (Figure 5). No differences in expression levels of adult (BHMT) or fetal Leydig cell markers (CRHR1) were detected in the present study (Tables 3 and 4). Instead, lower expression of INSL3, biomarker for Leydig cell functional capacity that has been associated with testicular descent [51] and support of gametogenesis [52] was confirmed in the IC‐LT group (FC ≥ 12‐fold; Table 3) and corroborated a reported decrease in INSL3 protein level in the testes of IC [22]. As the expression level of INSL3 had a high correlation with the Leydig cells N:C ratio, testosterone concentration, GTI, TI, VGI, and BGI (Figure 5), and was also proposed as an accurate and robust circulating biomarker for Leydig cell functional capacity in men [53], its suitability was additionally validated on samples derived from previous immunocastration studies [20]. We confirmed lower INSL3 expression in all successfully immunocastrated animals, irrespective of age or vaccination protocol (Table 4). Furthermore, neutralization by passive immunization reduced testis weight and sperm concentration by inducing germ cell apoptosis [54]. Recently, INSL3 monitoring was proposed for early detection of Leydig cell insufficiency in primary hypogonadism [53, 55] and evidence at the transcription level is provided for its suitability to confirm immunocastration‐induced hypogonadism in boars. Unlike testosterone, which fluctuates with puberty and age, INSL3 concentrations rise post‐puberty and remain stable in adulthood [56], suggesting it has potential as a reliable biomarker, with the need to define thresholds and reference ranges in future studies in boars.

## 5 Conclusion

Immunocastration is very effective in young (prepubertal) pigs, whereas pubertal boars had variable responses to immunocastration, irrespective of vaccination protocol. Testicular function had a nadir 4 week after GnRH suppression, with individual recovery at 8 and 12 week after a booster, probably influenced by LHCGR expression.

Therefore, when a two‐dose vaccination protocol is applied to various categories of pigs, for example, pigs fattened to a higher age and weight, young boars eliminated after a performance test, or adult boars eliminated from an artificial insemination center, the strategy of a late booster (4 week before slaughter) should be followed to ensure the best vaccination outcome. However, in production systems where swine are destined for high‐value meat products, androgen deficiency should be confirmed or a second booster given if economically feasible to eliminate the potential risk of boar taint. GTI, TI, Leydig cells N:C ratio and INSL3 transcript level were identified as reliable end points to evaluate the success of immunocastration. Considering that INSL3 expression directly correlated with the morphology and functionality of Leydig cells, it can be considered a prospective biomarker for immunocastration in boars. However, thresholds and reference ranges for circulating INSL3 should be established in future studies.

## Author Contributions

*Conceptualization*: Nina Batorek Lukač, Milka Vrecl, Martin Škrlep. *Methodology*: Nina Batorek Lukač, Gregor Fazarinc, Martin Škrlep, Milka Vrecl, Klavdija Poklukar. *Investigation*: Nina Batorek Lukač, Gregor Fazarinc, Martin Škrlep, Klavdija Poklukar, Petra Ferjan, Milka Vrecl. *Writing – Original Draft Preparation*: Nina Batorek Lukač. *Writing – Review & Editing*: Nina Batorek Lukač, Gregor Fazarinc, Volker Stefanski, Petra Ferjan, Klavdija Poklukar, Martin Škrlep, Milka Vrecl. *Funding Acquisition*: Martin Škrlep, Nina Batorek Lukač, Volker Stefanski, Milka Vrecl.

## Ethics Statement

According to Directive 2010/63/EU (2010) 119 and decision of Ethical Committee of the Veterinary Faculty, University of Ljubljana (decision No. 5‐4‐2018/5), the study was not subject to ethical protocols. The Institute of Preclinical Sciences, Veterinary Faculty of the University of Ljubljana is approved by the Veterinary Administration of the Republic of Slovenia for collection and use of animal by‐products (regulation (EC) No 1069/2009) for research purposes (permit No. SI‐B‐07‐22‐07). The use of animal by‐products (i.e., testis samples for research purposes) was also approved by the Veterinary Administration of the Republic of Slovenia (U34453‐1/2024/5).

## Conflicts of Interest

The authors declare no conflicts of interest.

## Supporting information




**Table S1**: Effect of the clusters vs. controls on end points related to sexual development and response to immunocastration (n = 23).
