# Supplementary material for: INSL3 Expression in Leydig Cells is a Biomarker for Immunocastration in Boars: Transcriptional Evidence
Source: Andrology. 2025 Oct 21;14(3):782–95. doi: 10.1111/andr.70136 (PMC12917571; doi:10.1111/andr.70136)
Supplement: Supplementary file 1 — Table S1: Effect of the clusters vs. controls on end points related to sexual development and response to immunocastration (n = 23). [file ANDR-14-782-s001.docx]

**Supplementary material**

**Supplementary Table 1.** Effect of the clusters *vs.* controls on end points related to sexual development and response to immunocastration (n=23).

| **End point** | **IC-LT^1^ (n=6)** | **IC-HT^1^ (n=11)** | **EM**  **(n=6)** | **P-value^2^** |
| --- | --- | --- | --- | --- |
| Genital tract index^3^ (%) | 0.29 ^a^ [0.18] | 0.58 ^b^ [0.21] | 0.63 ^b^ [0.13] | 0.005 |
| Testis index^4^ (%) | 0.37 ^a^ [0.20] | 0.79 ^b^ [0.20] | 0.84 ^b^ [0.10] | 0.012 |
| Vesicular gland index^5^ (%) | 0.05 ^a^ [0.07] | 0.22 ^b^ [0.13] | 0.29 ^b^ [0.12] | 0.015 |
| Bulbourethral glands index^6^ (%) | 0.09 ^a^ [0.12] | 0.16 ^b^ [0.05] | 0.21 ^b^ [0.04] | 0.005 |
| Colour of testicular tissue CIE^7^ L | 57.3 ^b^ [5.4] | 53.9 ^ab^ [5.1] | 51.1 ^a^ [3.4] | 0.022 |
| Colour of testicular tissue CIE^7^ a | 14.0 [5.0] | 16.3 [1.9] | 17.8 [1.8] | 0.107 |
| Colour of testicular tissue CIE^7^ b | 9.3 [3.0] | 7.5 [1.2] | 6.8 [1.2] | 0.087 |
| Nucleus to cytoplasm ratio in Leydig cells^8^ | 0.42 ^c^ [0.22] | 0.22 ^b^ [0.05] | 0.21 ^a^ [0.04] | 0.002 |
| GnRH antibody binding (%) | 47.1 ^c^ [4.3] | 32.9 ^b^ [ 11.7] | 7.8 ^a^ [0.0] | < 0.001 |
| Plasma testosterone (ng/mL) | 0.56 ^a^ [0.5] | 7.04 ^b^ [4.4] | 10.5 ^b^ [5.2] | < 0.001 |
| Fat androstenone (µg/g liquid fat) | 0.24 ^a^ [0.0] | 0.82 ^b^ [2.4] | 2.4 ^b^ [1.8] | 0.002 |

IC, immunocastrated male pigs; EM, entire male pigs; CIE, Commission Internationale de l'Elcairage.

^1^ Vaccination with Improvac ^®^ (2 mL, s.c. application, Zoetis using various intervals between booster and slaughter: 4, 8 or 12 wk. Original data were subjected to principal component analysis (ncf. Fig. 2) on selected variables (n =13) related to sexual development and response to immunocastration, followed by hierarchical clustering (ncf. Fig.3) to divide IC as the one clustered with EM (characterized by plasma testosterone > 1 ng/mL and thus labeled as IC-HT) and the remaining group of IC (with plasma testosterone < 1 ng/mL; labeled IC-LT).

^2^ Wilcoxon rank-sum test with Bonferroni adjustment.

^3^ Calculated as the genital tract weight (weight of the pelvic part of the genital tract, together with the accessory glands and emptied bladder) divided by warm carcass weight x 100.

^4^ Calculated as the testis weight (weight of the right and left testes with the epididymis included) divided by warm carcass weight x 100.

^5^ Calculated as the vesicular gland weight divided by warm carcass weight x 100.

^6^ Calculated as bulbourethral gland weight (weight of both bulbourethral glands and urethra) divided by warm carcass weight x 100.

^7^ CIE L, a, b color space; L=lightness, higher number denotes a lighter color; a=redness, higher number denotes a redder color; b=yellowness, higher number denotes a yellower color.

^8^ Calculated as the Leydig cell's nucleus area divided by the cytoplasm area.
